# 3D Neuromorphic Hardware with Single Thin‐Film Transistor Synapses Over Single Thin‐Body Transistor Neurons by Monolithic Vertical Integration

**DOI:** 10.1002/advs.202302380

**Published:** 2023-09-15

**Authors:** Joon‐Kyu Han, Jung‐Woo Lee, Yeeun Kim, Young Bin Kim, Seong‐Yun Yun, Sang‐Won Lee, Ji‐Man Yu, Keon Jae Lee, Hyun Myung, Yang‐Kyu Choi

**Affiliations:** ^1^ School of Electrical Engineering Korea Advanced Institute of Science and Technology (KAIST) 291 Daehak‐ro, Yuseong‐gu Daejeon 34141 Republic of Korea; ^2^ SK Hynix Inc. Icheon 17336 Republic of Korea; ^3^ Department of Materials Science and Engineering Korea Advanced Institute of Science and Technology (KAIST) 291 Daehak‐ro, Yuseong‐gu Daejeon 34141 Republic of Korea

**Keywords:** neuromorphic hardware, single thin‐film transistor synapse (1TFT‐synapse), single transistor neuron (1T‐neuron), spiking neural network (SNN), vertical 3D integration

## Abstract

Neuromorphic hardware with a spiking neural network (SNN) can significantly enhance the energy efficiency for artificial intelligence (AI) functions owing to its event‐driven and spatiotemporally sparse operations. However, an artificial neuron and synapse based on complex complementary metal‐oxide‐semiconductor (CMOS) circuits limit the scalability and energy efficiency of neuromorphic hardware. In this work, a neuromorphic module is demonstrated composed of synapses over neurons realized by monolithic vertical integration. The synapse at top is a single thin‐film transistor (1TFT‐synapse) made of poly‐crystalline silicon film and the neuron at bottom is another single transistor (1T‐neuron) made of single‐crystalline silicon. Excimer laser annealing (ELA) is applied to activate dopants for the 1TFT‐synapse at the top and rapid thermal annealing (RTA) is applied to do so for the 1T‐neuron at the bottom. Internal electro‐thermal annealing (ETA) via the generation of Joule heat is also used to enhance the endurance of the 1TFT‐synapse without transferring heat to the 1T‐neuron at the bottom. As neuromorphic vision sensing, classification of American Sign Language (ASL) is conducted with the fabricated neuromorphic module. Its classification accuracy on ASL is ≈92.3% even after 204 800 update pulses.

## Graphical abstract

A neuromorphic module composed of synapses over neurons is demonstrated by monolithic vertical integration. The synapse at top is a single thin‐film transistor (1TFT‐synapse) made of poly‐crystalline silicon film and the neuron at bottom is another single transistor (1T‐neuron) made of single‐crystalline silicon. As neuromorphic vision sensing, classification of American Sign Language (ASL) is conducted with the fabricated neuromorphic module.

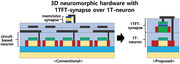

## 1 Introduction

Software‐based artificial neural networks (ANNs) have been widely used to conduct various intelligent tasks, such as pattern classification, image classification, and to design circuits.^[^ ^1^, ^2^, ^3^ ^]^ However, increased energy consumption, ascribed to the massive amount of data transmission between the processors and memory components in traditional von Neumann computing, has been a chronic problem.^[^ ^4^ ^]^ As an alternative to von Neumann computing, neuromorphic computing, which mimics the functioning of the brain, has attracted attention.^[^ ^5^, ^6^, ^7^ ^]^ With the mimicry of a biological neural network with a spiking neural network (SNN) that transmits information with spikes, energy‐efficient processing is possible due to its event‐driven and spatiotemporally sparse operations.

**Figure 1:**
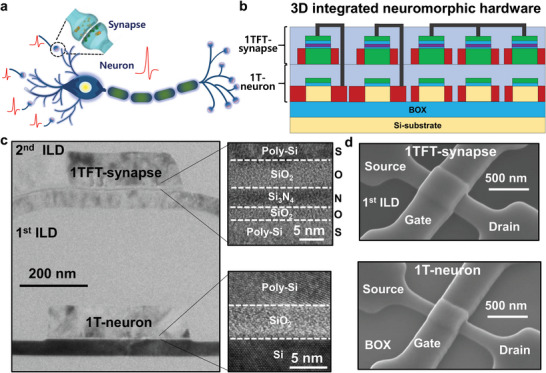
3D‐integrated neuromorphic hardware composed of single transistor‐neuron (1T‐neuron) at the top and single thin‐film transistor‐synapse (1TFT‐synapses) at the bottom. a) Schematic illustration of biological neurons and synapses in the human brain, which is composed 10^11^ neurons and 10^15^ synapses. b) Schematic illustration of 3D‐integrated neuromorphic hardware with superjacent 1TFT‐synapses over underlying 1T‐neurons with other CMOS interface circuits. c) Transmission electron microscope (TEM) images of the 3D‐integrated 1T‐neuron and 1TFT‐synapse. The 1T‐neuron has a single‐crystalline Si channel and SiO_2_ gate dielectric, while the 1TFT‐synapse has a poly‐crystalline Si channel and SiO_2_/Si_3_N_4_/SiO_2_ gate dielectrics. d) Scanning electron microscope (SEM) images of the 3D‐integrated 1T‐neuron and 1TFT‐synapse.

Neuromorphic hardware is composed of two elementary components: neurons and synapses, akin to a biological neural network, as illustrated in **Figure** **1**a. The neuron operation produces a spike signal as the output voltage when the input current exceeding a certain threshold is transmitted from the artificial synapses. The synapse operation memorizes and modulates the weight between two linked neurons. This feature is referred to as synaptic plasticity. Noting that there are ≈10^11^ neurons and 10^15^ synapses in the brain, it is difficult directly to mimic a brain system with neuromorphic hardware. One of technical challenges is to realize a high packing density of the neurons and synapses with low energy consumption. This stringent demand becomes more critical when applying neuromorphic hardware to edge computing, such as on a mobile device and on an Internet of Things (IoT) system. A conventional neuron and synapse based on a complex circuit composed of complementary metal‐oxide‐semiconductor (CMOS) transistors and capacitors have limited scalability and relatively low energy efficiency.^[^ ^8^, ^9^ ^]^

To overcome the limitations of circuit‐based neurons and synapses, device‐level neurons and synapses based on a two‐terminal memristor or a three‐terminal transistor have been developed with their own advantages. The memristor‐based neuron has the advantages of a smaller size and lower operation voltage.^[^ ^10^, ^11^ ^]^ In contrast, the transistor‐based neuron harnesses a few merits, including better CMOS compatibility with other indispensable control circuits and greater functionality, such as inhibition and homeostasis, due to its three‐terminal operation.^[^ ^12^, ^13^ ^]^ Like the memristor‐based neuron, a memristor‐based synapse with two terminals is attractive given its smaller size and lower operation voltage.^[^ ^14^, ^15^ ^]^ In contrast, a transistor‐based synapse with three terminals is favorable for its better stability, higher selective weight update without a selector, and wider conductance range to allow multiple states.^[^ ^16^, ^17^, ^18^ ^]^

Typically, neurons and synapses are studied individually. However, the realization of neuromorphic hardware strictly requires a hybrid form of integration. Recently, the integration for full neuromorphic hardware has been assessed. For example, a fully memristive neural network that integrates memristor‐based artificial neurons and synapses with a single crossbar array on a coplanar surface in a 2D setup was reported.^[^ ^19^, ^20^, ^21^ ^]^ In addition, our group demonstrated the co‐integration of single‐transistor‐based artificial neurons and synapses on a 2D plane created via 100% CMOS fabrication by laterally positioning the neurons and synapses.^[^ ^22^ ^]^ Co‐integration was possible in this case on a coplanar surface because the transistor neurons and synapses used are homologs to each other, i.e., they have the same structure but work differently.

In terms of the packing density, vertical integration with a structure of one over the other is preferred. Because synapses greatly outnumber neurons in the brain, many layered synapses need to be integrated over neurons, in addition to the fact that complex routing is necessary to form a neural network.^[^ ^23^, ^24^, ^25^, ^26^ ^]^ Commercial monolithic 3D stacking technology with a trillion transistors, currently being applied to a 3D vertical NAND flash architecture (3D VNAND) in which vertically stacked memory cells are positioned over periphery circuits, can feasibly support the idea of 3D neuromorphic hardware with the structure of “synapses over neurons” for a brain‐level neuromorphic system.

On the one hand, there have been a few attempts to integrate memristor‐based synapses over CMOS control circuits. In terms of manufacturing, these efforts have both strengths and weaknesses. One advantage is that the underlying CMOS circuits are scarcely influenced by the post‐processing temperature when creating the superjacent synapses due to the inherent low‐temperature fabrication process. However, a disadvantage is that the manufacturing of the synapse to create cross‐lined top and bottom electrodes can impose an additional burden on the metal interconnections by wiring each synapse at the back‐end‐of‐line (BEOL) stage. From an operational point of view, none of these methods can integrate leaky integrate‐and‐fire (LIF) neuron devices for a SNN.^[^ ^27^, ^28^, ^29^ ^]^ On the other hand, another approach can be used to integrate CMOS‐based synapses over CMOS‐based neurons. Although vertically stacked neuromorphic CMOS devices are advantageous given their high packing density and CMOS compatibility, they are vulnerable to high‐temperature processes.^[^ ^30^, ^31^ ^]^ When the thermal annealing temperature exceeding 1000 °C is applied to activate the dopants in the synapse at the top, the neuron at the bottom can also be affected hence an undesirable outcome is made.

In this work, we demonstrate 3D neuromorphic hardware by vertically positioning synaptic transistors over neuron transistors by means of a step‐by‐step monolithic integration process, as shown in Figure 1b. Each synapse at the top is a single thin‐film transistor (1TFT‐synapse) consisting of poly‐crystalline silicon (poly‐Si) and charge trap nitride for a tunable synaptic weight. Every neuron at the bottom is another single transistor (1T‐neuron) composed of single‐crystalline silicon (sc‐Si) with a floating body (FB) on a silicon‐on‐insulator (SOI) wafer and thermally grown oxide. Excimer laser annealing (ELA) is adopted to activate the dopants selectively in the superjacent 1TFT‐synapse in the absence of any heat transfer to the underlying 1T‐neuron. The 1T‐neuron must be located at the bottom because the sc‐Si is necessary to enable a single transistor latch for the operation of the neuron. It also must be co‐integrated with other CMOS control circuits on the same plane at the same time to ensure fabrication simplicity. The preferred location for the 1TFT‐synapse is at the top because a poly‐Si channel is not problematic for synaptic operation. Another reason is that layer‐by‐layer integration is possible for multiplying stacked synapses with a poly‐Si channel, as in the abovementioned 3D VNAND.

Moreover, good endurance characteristics of the synapse, which is immune to iterative operational stresses, are essential for online training, which typically requires repeated weight updates.^[^ ^9^ ^]^ For a memristor‐based synapse, breakdown by the set‐stuck effect arising from repetitive cycles is a concern.^[^ ^32^, ^33^ ^]^ For a transistor‐based synapse, fatigue by iterative charge trapping and de‐trapping stemming from repeated operations is troublesome owing to the accumulated stress.^[^ ^34^, ^35^ ^]^ At worst, the device can be permanently destroyed. Thermal annealing is known to be effective to cure stress‐induced damage. To repair damaged devices, recovery with wafer‐scale ex situ annealing using a furnace or a process chamber can be done.^[^ ^36^ ^]^ As an alternative, chip‐scale in situ annealing with an embedded heater can be applied.^[^ ^37^ ^]^ These approaches are akin to global annealing because heat is propagated to all devices. In contrast to global annealing, local annealing is necessary for the selective curing of a damaged device. This is realized upon the use of Joule heat, which arises from the current flow via the inherent resistor of a 1TFT‐synapse. Herein, we demonstrate a local electro‐thermal annealing (ETA) method with Joule heat to recover a 1TFT‐synapse selectively without thermal deterioration of the 1T‐neurons at the bottom. With this local ETA applied to the top 1TFT‐synapse, it works even after 204 800 update pulses. Finally, we utilize the 3D neuromorphic hardware to classify the patterns of American Sign Language (ASL) to demonstrate the applicability to a dynamic vision sensor (DVS) system.

## 2 Results and Discussion

### 2.1 3D Integration of 1T‐Neurons and 1TFT‐Synapses

Figure 1b shows an illustration of the 3D neuromorphic hardware with 1TFT‐synapses over 1T‐neurons. Because the 1T‐neurons in this case have a conventional MOSFET structure using a sc‐Si channel, they can be co‐integrated with other CMOS circuitry for peripheral interfaces, clocking, and for input/output circuits on the same plane at the bottom.^[^ ^22^ ^]^ The detailed process flow is shown in Figure S1 (Supporting Information). A p‐type 8‐inch SOI wafer with a top sc‐Si layer of 55 nm was used as the starting material. For the fabrication of the 1T‐neurons at the bottom, sc‐Si was patterned for the channel, SiO_2_ was thermally grown for the gate dielectric, and n^+^ in situ‐doped poly‐Si was deposited for the gate electrode. Next, the gate was patterned and n^+^ heavy doping with ion implantation was applied for the source and drain (S/D) electrode. To activate the dopant, rapid thermal annealing (RTA) was applied for 3 s at 1000 °C. Due to the floating body (FB) effect in the 1T‐neuron, a single‐transistor latch (STL) is allowed and used for neuronal firing with a spike shape.

After the first inter‐layer dielectric (ILD) deposition over the 1T‐neurons, a thin film of undoped poly‐Si was deposited over the first ILD layer, after which patterning was done. As gate dielectrics for the 1TFT‐synapse, tunneling SiO_2_ (O), charge trap Si_3_N_4_ (N), and blocking SiO_2_ (O) were sequentially formed using low‐pressure chemical vapor deposition (LPCVD). Afterwards, n^+^ in situ doped poly‐Si was deposited for the gate electrode and was patterned. Later, n^+^ heavy doping with another ion implantation was applied for the S/D electrode. Because the n^+^ poly‐Si (S) gate covers the overlying ONO gate dielectrics on the undoped poly‐Si (S) channel, this structure has a SONOS configuration. Moreover, the ONO layer creates a quantum well with a finite depth to hold and emit electrons due to the intercalated charge trap nitride between the top blocking oxide and the bottom tunneling oxide. Hence, the 1TFT‐synapse can control the synaptic weight by trapping or de‐trapping electrons. For dopant activation in the S/D of the 1TFT‐synapse, ELA for optical annealing was applied instead of a thermal annealing method such as RTA. Therefore, the underlying 1T‐neurons and other transistors at the bottom are scarcely affected by any thermal stimulus due to the good thermal insulation of the first ILD. This type of light‐induced annealing to confine heat in a irradiated surface is preferred to minimize thermal interference in inter‐devices between the top and bottom level. Compared to the poly‐Si channel thickness of 50 nm and poly‐Si gate thickness of 100 nm and the first ILD layer with a thickness of 300 nm, the penetration depth of ≈6 nm stemming from its short wavelength is shallow.^[^ ^38^ ^]^ Thus, the heat induced by ELA cannot be transferred to other areas apart from the exposed S/D of the 1TFT‐synapse.

After the second ILD deposition step over the 1TFT‐synapses, metallization was applied to connect the underlying 1T‐neurons and the superjacent 1TFT‐synapse. Finally, 3D neuromorphic hardware with 1TFT‐synapses over 1T‐neurons was realized by monolithic integration, as shown in the transmission electron microscope (TEM) image in Figure 1c. The detailed fabrication processes are explained in the Experimental Section. As shown in Figure 1d, the nominal gate width (*W*_G_) and gate length (*L*_G_) are 200 and 400 nm, respectively, for the 1T‐neuron. For the 1TFT‐synapse, the nominal *W*_G_ and *L*_G_ values are 300 and 500 nm, respectively. The detailed device parameters are summarized in Table S1 (Supporting Information).

By changing the exposure energy of the excimer laser from 150 to 500 mJ cm^−2^ but with a fixed laser pulse width of 25 ns, the ELA condition was optimized with two strategies. The first is to find the proper condition under which to generate heat, which is a level similar to that of RTA. The second is to exploit the condition in which the underlying 1T‐neuron is thermally insulated from the heat created at the top. ELA was applied to the experimental group, whereas RTA of 2 s at 1000 °C was used for the control group. Each group has 15 samples. Compared to the annealing effects by RTA, those by ELA were verified by one electrical instance and two physical instances. As shown in Figure S2 (Supporting Information), three representative device parameters, the threshold voltage (*V*_T_), the subthreshold swing (*SS*), and the on‐state current (*I*_ON_), were compared between the two groups. In terms of the electrical evidence, they were comparable to each other. As shown in the TEM images in Figure S3 (Supporting Information) as direct physical evidence, both poly‐Si films were recrystallized from an amorphous phase, which was caused by a heavy dose of ion implantation for the S/D of the 1TFT‐synapse. As indirect physical evidence of recrystallization, optical images were also compared. Their colors look very similar, as shown in Figure S4 (Supporting Information). Based on these observations, ELA activated dopants as RTA did.

Despite the fact that heat induced by the ELA is high enough to recrystallize the amorphized S/D silicon film as well as to activate the dopants, it should not be transferred from the top level to the bottom level through the first ILD. To verify this, thermal simulations were conducted with the aid of a 3D thermal simulator (COMSOL). As shown in Figure S5a (Supporting Information), the heat distribution profile across the 3D neuromorphic hardware along the vertical direction was plotted by reflecting each film thickness and the corresponding temperature‐dependent parameters.^[^ ^39^ ^]^ Referring to Figure S5b (Supporting Information), the top poly‐Si film was heated to 1172 °C with an exposure energy of 200 mJ cm^−2^. This was high enough to recrystallize the poly‐Si film but low enough to avoid the melting of the Si. In contrast, the bottom poly‐Si film was sustained at a low temperature below 50 °C, which cannot influence the device characteristics of the underlying 1T‐neurons.

On the other hand, ELA at 300 mJ cm^−2^ heated the top poly‐Si film to 1563 °C, which is higher than the melting temperature of Si. At a higher activation rate, more energy is preferred unless the heat can destroy the device. Therefore, we adopted 200 mJ cm^−2^ for the optimal condition. As indirect electrical proof of the ELA, the transfer characteristics of the drain current versus gate voltage (*I*_D_–*V*_G_) and the output characteristics of the drain current versus the drain voltage (*I*_D_–*V*_D_) of the fabricated 1T‐neuron were measured. These values were then compared before and after ELA for the superjacent 1TFT‐synapses. They were found to be nearly identical, as shown in Figure S6 (Supporting Information). It should also be noted that the 1T‐neurons at the bottom were already annealed by the pre‐applied RTA prior to the first ILD deposition. The instantaneous ELA and the thermal insulation of the first ILD inhibit heat transfer from the top level to the bottom level.

### 2.2 Decoupled Characteristics of Superjacent 1TFT‐Synapses and Underlying 1T‐Neurons

**Figure 2:**
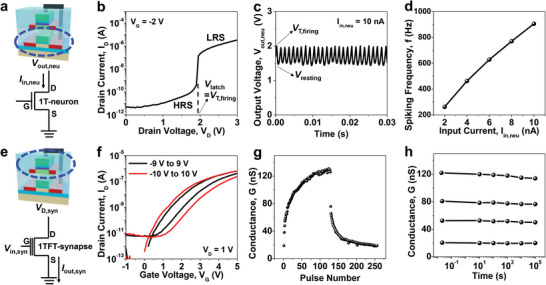
Measured characteristics of the decoupled 1T‐neuron (a,b,c,d) and 1TFT‐synapse (e,f,g,h). a) Cross‐sectional schematic of the 1T‐neuron with the corresponding symbolic representation to show the current input (*I*_in,neu_) to the drain and the voltage output (*V*_out,neu_) from the same drain. b) Neuronal output characteristic (*I*_D_–*V*_D_) of the fabricated 1T‐neuron. Abrupt threshold switching occurs at the latch‐up voltage (*V*_latch_) for the firing operation. c) Spiking characteristics (*V*_out,neu_‐*t*) of the fabricated 1T‐neuron. d) Spiking frequency (*f*) as a function of *I*_in,neu_ with the typical property of a LIF neuron. e) Cross‐sectional schematic of the 1TFT‐synapse with the corresponding symbolic expression to show the voltage input (*V*_in,syn_) to the gate and the current output (*I*_out,syn_) from the source. f) Neuronal transfer characteristic (*I*_D_‐*V*_G_) of the fabricated 1TFT‐synapse with hysteresis caused by charge trapping and detrapping in Si_3_N_4_. g) Potentiation and depression (P/D) characteristics of the fabricated 1TFT‐synapse with 128 conductance states in each case. h) Retention characteristics of the fabricated 1TFT‐synapse.

The *I*_D_‐*V*_D_ characteristics of the fabricated underlying 1T‐neuron (**Figure** **2**a) are plotted in Figure 2b. When *V*_D_ is lower than the latch‐up voltage (*V*_latch_), *I*_D_ does not flow because the 1T‐neuron is in a high‐resistance state (HRS). However, when *V*_D_ exceeds *V*_latch_, *I*_D_ flows abruptly given its low‐resistance state (LRS). *V*_latch_ is defined as the critical voltage to trigger the abovementioned STL, causing *I*_D_ to increase abruptly, *i*.*e*., an immediate change from a HRS to a LRS.^[^ ^40^ ^]^ This abrupt threshold switching is analogous to the firing in a biological neuron. In addition, *V*_latch_ corresponds to the firing threshold voltage (*V*_T,firing_). The charging and discharging process is repeated as long as a constant input current (*I*_in,neu_) is applied to the drain electrode of the 1T‐neuron. Figure 2c shows the neuronal spiking characteristics, represented by the oscillating output voltage (*V*_out,neu_) versus the time. The charging process equivalent to “integrating” is continued, gradually filling the parasitic drain capacitor of the 1T‐neuron; however, a discharging process identical to “firing” suddenly occurs via the abrupt threshold switching. The top peak is *V*_T,firing_ and the bottom peak is the resting voltage (*V*_resting_), as shown in Figure 2c. As shown in Figure 2d, a spiking frequency (*f*) increases as *I*_in,neu_ increases, representing a typical property of a leaky integrate‐and‐fire (LIF) neuron. Given this property, more spiking is generated when more signals are transmitted from previously activated synapses. Energy consumption is crucial for low‐power neuromorphic computing. The estimated amount of neuron energy consumed per spike (*E*_neuron_/spike), as extracted from $E_{\mathrm{neuron}}/\mathrm{spike}\;=I_{\mathrm{in},\mathrm{neu}}\;\int_{0}^{\frac{1}{f}}V_{\mathrm{out},\mathrm{neu}}dt$, is 19.3 pJ per spike at *I*_in,neu_ = 10 nA.^[^ ^22^ ^]^ As summarized in Table S2 (Supporting Information), this value is sufficiently low compared to other artificial neuron devices.

The stable operation of the 1T‐neuron is important because the damaged neuronal characteristics can degrade the system performance. To verify the endurance characteristics of the 1T‐neuron, spiking operations were repeated for 10 h while applying *I*_in_ of 10 nA. Considering that *f* of the 1T‐neuron is ≈1 kHz at *I*_in_ of 10 nA, it generates 3.6 × 10^7^ spikes during the endurance test. Figure S7a shows the transfer characteristics of the 1T‐neuron before and after 10 h of spiking operations. There is no notable degradation in subthreshold swing, threshold voltage, on‐state current, and off‐state current. It implies that an 1T‐neuron is scarcely damaged even after 3.6 ×  10^7^ spiking. Figure S7b (Supporting Information) compares the spiking characteristics (*V*_out_‐*t*) of the before and after 10 h of spiking operations. Figure S7c,d (Supporting Information) exhibit that *V*_T,firing_ and *f* were negligibly changed. These data support that the system performance would be maintained even after repetitive operations.

There are five representative features in a synaptic device. The first of these is the ability to allow multiple states. Figure 2e–h show the electrical properties of the superjacent 1TFT‐synapse. The synaptic weight can be controlled by changing the trapped charge density in the nitride of the SONOS structure. As shown by *I*_D_–*V*_G_ in Figure 2f, negative biasing of *V*_G_ causes a leftward shift of *V*_T_, akin to erasing during memory operation. This corresponds to potentiation during synaptic operation because the conductance (*G*) is increased due to the reduction of *V*_T_. This negative shift is attributed to the de‐trapping of charges in the nitride of the SONOS. Positive biasing of *V*_G_ induces a rightward shift of *V*_T_, analogous to programming during memory operation. This corresponds to depression during synaptic operation because *G* is decreased due to the increase in *V*_T_. These opposite shifts provoke hysteresis, which tends to be wider due to the larger |*V*_G_|. This potentiation and depression (P/D) characteristic, as represented by a change of *G* according to the number of applied pulses, is plotted in Figure 2g. Each of the 128 distinctive *G* outcomes in the P/D was produced with an identical pulse scheme to use a constant voltage amplitude (*V*_G_) and a fixed pulse width (*t*_pulse_), unlike other works that rely on a variable pulse scheme to utilize an incremental *V*_G_ or a wider *t*_pulse_. *V*_G_ of −12 V with *t*_pulse_ equal to 100 µs was used for potentiation and *V*_G_ of 9.3 V with *t*_pulse_ set to 10 µs was used for depression. *V*_G_ and *V*_D_ were set to 2.5 V and 1 V, respectively for the reading operation.

The second feature makes energy consumption per event (*E*_synapse_/event) as low as possible for a synaptic operation. It should be noted that *V*_D_ and the source voltage (*V*_S_) are grounded such that *I*_D_ cannot flow through the channel during the P/D. Therefore, *E*_synapse_ is dominated by the gate leakage current (*I*_G_) and can be estimated by *E*_synapse_/event = *I*_G_ *V*_G_ *t*_pulse_. Because *I*_G_ was in a range of a few pA, as shown in Figure S8 (Supporting Information), *E*_synapse_/event is 2.33 fJ/event for potentiation and 0.03 fJ per event for depression. These values are lower than the biological synapse energy of 10 fJ per event.^[^ ^41^ ^]^

Third is the retention characteristic. As shown in Figure 2h, distinctive states were sustained for longer than 10^5^ s due to the non‐volatile charge trapping in the nitride.^[^ ^42^ ^]^ The fourth feature is endurance, which is important for repetitive online learning. Fifth is recoverability from stress‐induced damage. A synapse will experience more frequent operation‐induced stress due to repeated synaptic updates and will undergo harsher voltage‐induced stress due to the high applied voltage. It should also be noted that the trapping and de‐trapping for synapse operation require a higher |*V*_G_| above 9 V to allow the tunneling of *I*_G_ intentionally via the ONO dielectrics, whereas the firing for the neuron operation requires a *V*_D_ value below 2 V. If possible, selective curing for a superjacent 1TFT‐syanspe can be done to improve endurance. Curing should selectively mitigate the fatigue or aging of a superjacent 1TFT‐synapse, which arises from the iterative electrical stresses, though it should not have any side effects on any underlying 1T‐neurons.

The stress‐induced damage was cured by ETA, which utilized the Joule heat generated in the device. This approach can selectively confine the Joule heat inside the target device. If a wafer‐scale global annealer such as furnace or RTA chamber is used, the well‐optimized thermal budget for an undamaged synapse or underlying 1T‐neurons can become overloaded, in turn causing the corresponding pre‐designed electrical behaviors to be deviated from the target values. Moreover, there is an upper limit of the allowable maximum temperature without the melting of the metal silicide or metal interconnections used. To generate Joule heat inside the 1TFT‐synapse, punchthrough current (*I*_punch_), which flows through n^+^ S via the undoped poly‐Si channel to n^+^ D, was utilized.^[^ ^43^, ^44^ ^]^ Once more, 3D thermal simulations with the aid of the commercial simulator (COMSOL) were conducted to analyze the temperature (*T*) distribution profile and to optimize the condition of the Joule heat.

**Figure 3:**
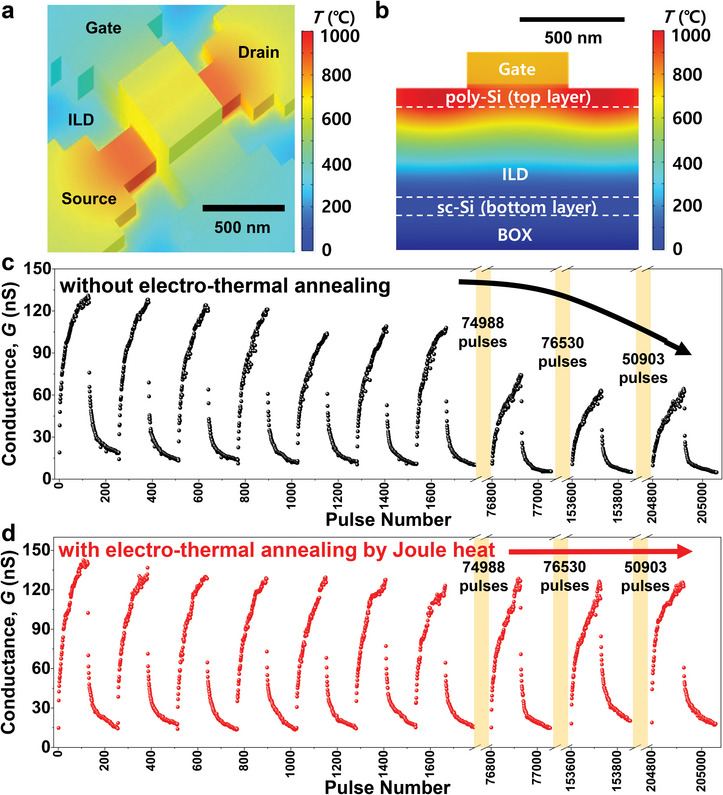
Improved endurance by electro‐thermal annealing with Joule heat. a) Heat distribution profile in the 1TFT‐synapse obtained from 3D thermal simulation. Joule heat is generated by the internal punchthrough current (*I*_punch_). b) The heat distribution profile along the vertical direction shows that the generated heat is not transferred to the bottom layer. Localized ETA to cure the fatigue of a 1TFT‐synapse does not affect the underlying 1T‐neuron. c) Degraded P/D characteristics caused by iterative operations of the 1TFT‐synapse. d) Cured P/D characteristics of the 1TFT‐synapse by ETA after fatigue arising from iterative operations.

When *I*_punch_ of less than 105 µA was applied, the induced *T* near the interface between the ONO gate dielectrics and the undoped poly‐Si channel was only 512 °C, which is not high enough to assist the thermal curing process, as shown in Figure S9a (Supporting Information). Otherwise, when *I*_punch_ exceeds 165 µA, the induced *T* reached 1415 °C, which is above the melting point of poly‐Si. Thus, the optimal *T* for curing by ETA is between these two values. When *I*_punch_ is 135 µA, *T* reached 833 °C, which is high enough to cure the stress‐induced damage on the gate dielectrics, as shown in **Figure** **3**a.^[^ ^37^, ^44^ ^]^ Although a high *T* is induced in the poly‐Si at the top, current induced heat is scarcely transferred to the sc‐Si at the bottom due to the good thermal insulation of the first ILD layer, as shown in Figure 3b and Figure S9b (Supporting Information). This facilitates the repair of the damage of the superjacent 1TFT‐synapse selectively and improves the endurance characteristics without affecting the underlying 1T‐neurons. Based on these results, it is inferred that a two‐level neuromorphic module of “synapses over neurons” is attractive not only for the resulting good packing density but also for long‐term endurance with selective curing while preventing thermal disturbances.

As shown in Figure 3c, the P/D characteristics were gradually degraded, *i*.*e*., the range of the *G* values was reduced owing to stress‐induced damage such as trap generation in the gate dielectric.^[^ ^35^ ^]^ Note that Fowler‐Nordheim (F‐N) stress induced by repetitive programming and erasing in SONOS flash memory degrades the reading current, which is related to *G* during the synaptic operation.^[^ ^44^ ^]^ As shown in Figure 3d, the endurance characteristics were appreciably improved with periodic ETA in each case after 2580 weight updates. Figure S10a (Supporting Information) compares the P/D curves before and after ETA. As expected, the range of *G* is broadened after ETA. Figure S10b (Supporting Information) compares the *I*_D_–*V*_G_ plots before and after ETA. The subthreshold swing (*SS*) of the superjacent 1TFT‐synapse was improved by 32.0% and the corresponding on‐state current (*I*_ON_) was enhanced by 50.7%. As shown in Figure S11 (Supporting Information), however, the spiking characteristics of the underlying 1T‐neuron were not changed after ETA was applied to the superjacent 1TFT‐synapse. This outcome confirms that ETA cured the synaptic fatigue selectively but did not change the neuronal characteristics.

By the way, it is necessary to evaluate energy consumption (*E*_ETA_) for ETA. The applied *I*_punch_ and voltage (*V*_ETA_) for ETA were 165 µA and 18 V, respectively, while a pulse width (*t*_ETA_) of the *V*_ETA_ was 1 ms. Therefore, the *E*_ETA_ can be calculated as 2.97 µJ with *I*_punch_ *V*_ETA_ *t*_ETA_. This *E*_ETA_ is much larger than spiking energy of 19.3 pJ per spike by an 1T‐neuron and weight update energy of 0.03 fJ per event in an 1TFT‐synapse. However, it may not be a concern because ETA to enhance the endurance of the 1TFT‐synapse is not frequently applied. Furthermore, down‐scaling of a gate length (*L*_G_) and gate width (*W*_G_) of an 1TFT‐synapse can help lower the *E*_ETA_ because the demanded *I*_punch_, *V*_ETA_, and *t*_ETA_ are accordingly reduced.^[^ ^44^, ^45^ ^]^

Table S3 (Supporting Information) compares the synaptic characteristics among previously reported synaptic devices with 3D stacking and those in this work.^[^ ^26^, ^28^, ^29^ ^]^ There are two notable features in this work. The first is that it represents the first implementation of SNN by stacking synaptic devices onto LIF neurons. The second feature is the selectively curable endurance realized by ETA. Table S4 (Supporting Information) presents the device‐to‐device variation variability of 1T‐neurons and 1TFT‐synapses, which includes the average and standard deviation values of various device parameters. The electrical characteristics of 10 distinct 1T‐neurons and 1TFT‐synapses were measured. Notably, no significant variations were observed, thus facilitating large‐scale integration.

### 2.3 Coupled Characteristics of Superjacent 1TFT‐Synapses and Underlying 1T‐Neurons

**Figure 4:**
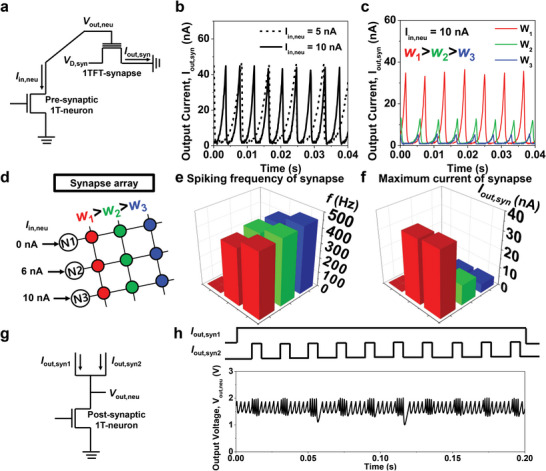
Coupled neuromorphic operation of 1TFT‐synapses at the top and 1T‐neurons at the bottom. a) Vertical hybrid of a pre‐synaptic 1T‐neuron and 1TFT‐synapse. b) Measured *I*_out,syn_ from a 1TFT‐synapse versus time for various values of *V*_out,neu_ from the 1T‐neuron. Here, *f* increases with a larger *I*_in,neu_. c) Measured *I*_out,syn_ according to the synaptic weight. *I*_out,syn_ increases with a greater synaptic weight. d) Array structure composed of the pre‐synaptic 1T‐neurons and 1TFT‐synapses. e) Extracted *f* from each 1TFT‐synapse cell. f) Extracted maximum value of *I*_out,syn_ from each 1TFT‐synapse cell. g) Post‐synaptic 1T‐neuron collecting and receiving *I*_out,syn_ outputs from two different 1TFT‐synapses. h) *V*_out,neu_ from the post‐synaptic 1T‐neuron depending on the two *I*_out,syn_ signals. *f* is higher when two signals are applied coincidently, thus demonstrating the function of spatio‐temporal coincidence detection.

To realize an artificial neural network, the cooperative properties of the 1T‐neurons and the 1TFT‐synapses should be investigated in addition to their individual properties. **Figure** **4**a shows a circuit diagram for the cooperation between a pre‐synaptic 1T‐neuron and a 1TFT‐synapse. As long as a constant *I*_in,neu_ is fed to the drain of the pre‐synaptic 1T‐neuron, an oscillating *V*_out,neu_ was emitted from it. Then, this *V*_out,neu_ was applied to the gate of the 1TFT‐synapse as an input signal. Finally, the output current from the 1TFT‐synapse (*I*_out,syn_) reflecting the synaptic weight, which would be transmitted to a post‐synaptic 1T‐neuron, flowed through the 1TFT‐synapse. As shown in Figure 4b, the spiking frequency (*f*) of *I*_out,syn_ increases as *I*_in,neu_ increases.

In addition, the maximum *I*_out,syn_ becomes larger as the synaptic weight (*w*_i_) increases, as shown in Figure 4c. This feature is a desired characteristic for the connection between the pre‐synaptic neuron and the synapse. Here, *I*_out,syn_ was measured from the array depicted in Figure 4d. In this case, a different *I*_in,neu_ was applied to each 1T‐neuron in a row and a different *w*_i_ was emitted from each 1TFT‐synapse in a column. Figure 4e,f show the measured *f* and maximum *I*_out,syn_ from each 1TFT‐synapse, respectively. It could be verified that the higher value of *f* is produced by the larger *I*_in,neu_ from the pre‐synaptic 1T‐neuron and that the larger *I*_out,syn_ is generated by the stronger *w*_i_ from the 1TFT‐synapse.

Figure 4g,h shows the operation of a post‐synaptic 1T‐neuron when *I*_out,syn_ is transferred from the 1TFT‐synapses. As illustrated in Figure 4g, *I*_out,syn_ from the 1TFT‐synapses is applied to the drain electrode of the post‐synaptic 1T‐neuron. Considering that more than a few hundred synapses are connected to each neuron in the brain, the collected current from many synapses with various values of *w*_i_, assuming that all are to be transmitted to a neuron, is too large to be adapted to a proper value of *I*_in,neu_. Hence, *I*_out,syn_ must be reduced. A poly‐Si channel with low mobility and a narrow channel width like our 1TFT‐syanpse is preferred to satisfy this demand. More aggressively, an extra circuit component may be needed when numerous synapses, i.e., more than ten thousand, are linked to a neuron. In such a case, the current mirror depicted in Figure S12 (Supporting Information)**_,_** which is co‐integrated with the 1T‐neuron prior to the first ILD deposition, is necessary to reduce the level of *I*_out,syn_ further.^[^ ^22^ ^]^ As an experimental example of cooperation between the post‐synaptic 1T‐neuron and the 1TFT‐synapses, a coincidence detection process was utilized to depict the spatio‐temporal neural computation. In biology, coincidence detection, referring to the encoding of information by identifying the occurrences of input signals that are spatially distributed but close in time, is used as an important neural computation process in visual and auditory systems.^[^ ^46^, ^47^, ^48^ ^]^ As shown in Figure 4h, when the two inputs of *I*_out,syn1_ and *I*_out,syn2_ were applied together, *f* was increased because the summed *I*_in,neu_ applied to the post‐synaptic 1T‐neuron was increased. In light of this, it is feasible to ascertain whether two inputs are synchronized.

By the way, the number of synapses significantly exceeds the number of neurons in the real neuromorphic system. This indicates that the area of the synapse array is much larger than that of the neurons with supportive circuits, causing challenges in layout. To enhance the layout efficiency of the synapses, a 3D vertical NAND (3D VNAND) structure can be applied.^[^ ^49^, ^50^ ^]^ It should be noted that the peri‐under‐cell (PUC) or cell‐on‐peri (COP) structure used in 3D VNAND, where their cells are fabricated on the peripheral circuits using monolithic 3D integration, is similar to our 3D neuromorphic hardware.^[^ ^51^ ^]^

### 2.4 Neuromorphic Vision Sensing with a Spiking Neural Network

In addition to face and pattern recognition abilities, neuromorphic vision sensing (NVS) is promising for gesture classification with reduced power consumption, which is attributed to fewer redundant data transfers and information processing with the SNN.^[^ ^52^, ^53^ ^]^ To confirm that the 3D‐integrated neuromorphic hardware can be applied to NVS, we performed two types of simulations. The first was a hardware‐based circuit simulation with the aid of SPICE for simple 3 × 3 kernel operation to recognize a letter pattern using a single‐layer SNN. The second was a software‐based neural network simulation with the aid of PyTorch for complex American Sign Language (ASL) classification using a spiking convolutional neural network (spiking‐CNN). Note that the kernel operation is a basic element of the spiking‐CNN.

Prior to the circuit simulation with SPICE, the 1T‐neuron must be modeled with sub‐elements. Thus, it was modeled with parallel connection consisting of a threshold switch and a parasitic capacitor.^[^ ^22^ ^]^ The measured *V*_T,firing_ and *V*_resting_ from Figure 2c for the threshold switch and the extracted *C*_par_ for the parasitic capacitor were reflected in the circuit simulations with SPICE. The 1TFT‐synapse was modeled with a three‐terminal MOSFET and the corresponding *V*_T_ was adjusted to control *w*_i_. The circuit for the 3 × 3 kernel operation was composed of nine input synapses corresponding to each of nine pixels and one output neuron, with a current mirror located between the synapses and the neuron to reduce *I*_out,syn_. The circuit of the 3×3 kernel is identical to the circuit schematic in Figure S12 (Supporting Information) except for the number of synapses. *V*_in,syn_ is applied to the gate of the 1TFT‐synapse to modulate *w*_i_, which is correlated with the pixel intensity. It became elevated at a higher pixel intensity level, as illustrated in Figure S13a (Supporting Information). As a simple example of letter pattern recognition, three different images including noisy signals were prepared. These three letter patterns were passed through three different kernels (“n”‐ kernel, “v”‐kernel, and “z”‐kernel). When the kernel matched the input image, a high *f* is produced at the corresponding output neuron. As shown in Figure S13b (Supporting Information), the “n”‐kernel generated the highest *f* for the noisy “n” pattern compared to *f* for the noisy “v” and “z” patterns. This outcome indicates that the “n”‐kernel successfully recognized the “n” image. In the same way, the “v”‐ and “z”‐kernels generated the highest *f* for the noisy “v” and “z” patterns, respectively.

Prior to the network simulation, a pre‐image processing step is necessary. Most of the image classification process is usually performed using a general image dataset collected with a standard frame‐based camera.^[^ ^13^, ^17^ ^]^ However, when using a general image dataset, image pixel values must be extracted in every frame and encoded in a spike form. Few difficulties arise when using that approach, *e*.*g*., the limited frame rate, high redundancy between frames, and high power consumption. Therefore, it is recommended to perform NVS using an image dataset obtained directly from a dynamic vision sensor (DVS). In particular, the ASL data were obtained by filming real sign language with a DVS.^[^ ^54^ ^]^ Because the ASL dataset has 24 classes (A‐Y, excluding J) and each letter consists of 4200 samples, the total number of samples is 100 800. It is important to show whether the proposed 3D‐integrated neuromorphic hardware can classify complicated ASL. Among the total of 100 800 samples, 84 000 samples were randomly selected for the training and the others were used for the testing.

**Figure 5:**
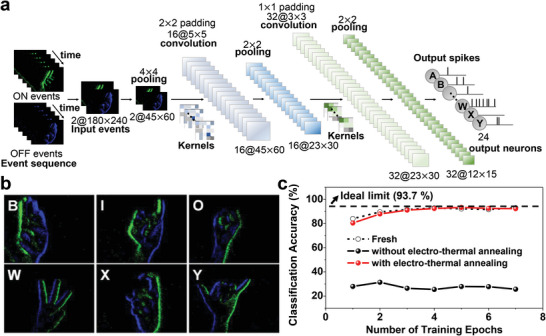
Neuromorphic vision sensing (NVS) to classify American Sign Language (ASL). a) Configuration of the spiking convolutional neural network (spiking‐CNN) for ASL classification. b) Examples of the ASL dataset obtained by filming actual sign language with the aid of a dynamic vision sensor (DVS). c) Classification accuracy according to the number of training epochs. Three measured synaptic P/D characteristics in three states: fresh, fatigued after 204 800 weight updates, and cured with ETA after 204 800 weight updates. The classification accuracy after curing is comparable to that in the fresh state.

**Figure** **5**a shows the spiking‐CNN configuration. A few exemplary gestures in ASL are shown in Figure 5b as examples. Each hand gesture has an image size of 180 × 240 pixels. The pixel values stored in ASL data were applied as inputs to the 1T‐neurons and 1TFT‐synapses. Then, LIF operations were performed by 1T‐neurons, and multiply‐and‐accumulate (MAC) calculations were conducted by 1TFT‐synapses, respectively. The spiking‐CNN consists of two spiking convolutional layers, three average pooling layers, and two fully connected layers, as summarized in Table S5 (Supporting Information). It should be noted that the measured characteristics of the underlying 1T‐neuron and the superjacent 1TFT‐synapse were reflected for the spiking‐CNN simulation to classify various hand gestures. For the neuron, the measured the spiking characteristics in Figure 2c were reflected, as mentioned above. For the synapse, the measured P/D characteristics in Figure 2g were reflected to model the conductance modulation using the following equation:

(1) $$G\;=\left\{\begin{array}{cc} {\left(\left[G_{\max}^{\alpha }-G_{\min}^{\alpha }\right]+G_{\min}^{\alpha }\right)}^{1/\alpha } & \mathrm{if}\;\alpha \neq 0\\ G_{\min}^{\alpha }\left(G_{\max}/G_{\min}\right) & \mathrm{if}\;\;\alpha =0 \end{array}\right.$$

where *α* is a parameter that controls the potentiation (*α*_pot_) or depression (*α*_dep_) and *η* is an internal variable ranging from 0 to 1.^[^ ^55^ ^]^ Extracted parameters of P/D characteristics for three cases (fresh, after 204 800 update pulses without ETA, after 204 800 update pulses with ETA) were summarized in Table S6 (Supporting Information). To train the network, backpropagation by means of spike layer error reassignment in time (SLAYER) was utilized.^[^ ^56^ ^]^ When using this method, the probability distribution function (PDF) of the state change was used for differentiating the spike function. Such a PDF change from *not‐fire* to *fire* or from *fire* to *not‐fire* decays exponentially according to the absolute value of the difference between the membrane potential and *V*_T,firing_. More details pertaining to the simulation are given in Note S1 (Supporting Information).

The classification accuracy of 93.0% was obtained with a fresh 1TFT‐synapse without cyclic degradation, as shown in Figure 5c. This outcome is close to the upper limit of 93.7%, which is achievable with perfectly linear P/D characteristics (*α*_pot_ = *α*_dep_ = 1) in the simulation. Successful classification is also confirmed by the Video S1 (Supporting Information), which visualizes the number of output spikes generated from each output neuron according to the time elapse. The corresponding output neuron matches the input image that generates the largest number of spikes. It should be emphasized that the classification accuracy falls to 31.4% after 204800 updates unless ETA is applied for recovery. In contrast, it only slightly decreases to 92.3% even after 204 800 updates with ETA. Finally, we demonstrated the long‐term durable NVS operation with the proposed 3D‐integrated neuromorphic devices with ETA.

## 3 Conclusion

In summary, we demonstrated 3D‐integrated neuromorphic hardware with a two‐level neuromorphic “synapse over neuron” structure. A superjacent poly‐crystalline Si thin‐film transistor for the synapse (1TFT‐synspse) was vertically positioned over an underlying single crystalline‐Si transistor for the neuron (1T‐neuron) by 3D monolithic integration. Thermal interference between the top‐level synapses and the bottom‐level neurons was avoided with the use of optical annealing with an excimer laser, which was used for dopant activation in the 1TFT‐synapses. After an examination of the individual characteristics of the 1T‐neuron and the 1TFT‐synapse, their cooperation characteristics were investigated to demonstrate spatio‐temporal neural computation capabilities. In addition, ASL classification was conducted to demonstrate the applicability of the 3D‐integrated neuromorphic hardware for neuromorphic vision sensing based on a dynamic vision sensor. Localized electro‐thermal annealing (ETA) using the Joule heat arising from the device itself was selectively applied to the superjacent 1TFT‐synapse to improve the endurance characteristics without affecting the underlying 1T‐neurons. As a result, American Sign Language (ASL) was successfully classified with the aid of ETA even after 204 800 updates pulses. The two most innovative aspects of this work, which are the 3D integration of 1TFT‐synapse over 1T‐neuron and local ETA with Joule heat, will pave the way toward the realization of highly scalable and durable neuromorphic hardware systems in the future.

## 4 Experimental Section

### Fabrication of the 3D‐Integrated 1T‐Neurons and 1TFT‐Synapses

A p‐type (100) SOI wafer with a buried‐oxide (BOX) thickness of 145 nm and a sc‐Si thickness of 55 nm was used as the starting substrate. First, the sc‐Si channel for the 1T‐neuron was patterned by photolithography and plasma etching, SiO_2_ was thermally grown to 5 nm for the gate dielectric, and an in situ‐doped n^+^ poly‐Si of 100 nm was deposited for the gate electrode. Afterwards, the gate was patterned by another photolithography and plasma etching, the region for the source and drain (S/D) was doped via arsenic implantation at 55 keV of energy with a dose of 5 × 10^15^ cm^−2^. Subsequently, RTA at 1000 °C was applied for 3 sec and tetraethyl orthosilicate (TEOS) of 300 nm was then deposited for the first ILD, which was used to isolate the underlying 1T‐neurons and the superjacent 1TFT‐synapse. Next, undoped poly‐Si of 50 nm for the 1TFT‐synapse over the first ILD was deposited and patterned by photolithography and etching. Tunneling SiO_2_ of 3 nm, charge trapping Si_3_N_4_ of 6 nm, and blocking SiO_2_ of 8 nm was sequentially deposited for the ONO gate dielectrics. For the gate electrode, another in situ‐doped n^+^ poly‐Si layer of 100 nm was deposited and patterned by photolithography and etching. Afterwards, another S/D doping was conducted with arsenic implantation with an energy of 70 keV and a dose of 5 × 10^15^ cm^−2^. For dopant activation, ELA was performed with an energy density of 200 mJ cm^−2^ for 25 ns. Finally, another TEOS layer of 300 nm was deposited for the second ILD, which was used to isolate the 1TFT‐synapses and uppermost metal interconnections. Metallization with Al of 1 µm was then used to connect the underlying 1T‐neurons and the superjacent 1TFT‐synapses. The maximum temperature applied to the underlying 1T‐neurons was 650 °C to deposit the ILD with LPCVD.

### Excimer Laser Annealing (ELA)

A XeCl excimer laser (Coherent, CompexPro205, λ = 308 nm) was used for dopant activation of the S/D in the superjacent 1TFT‐synapses. The ELA system was composed of a beam delivery apparatus, an attenuator, a square‐patterned mask, a homogenizer, and a xyz linear stage (Dukin, SLS‐200). It controls the irradiating fluence with a flat‐top square beam on the sample. The laser shot was scanned along a “zig‐zag” shaped pattern with an optimized energy density level.

### Joule Heat Annealing

An optimal condition to flow *I*_punch_ was explored to generate Joule heat, which can cure damage to a 1TFT‐synapse but does not have any effect on the underlying 1T‐neurons. In this case, *I*_punch_ of 135 µA was applied with *V*_G_ of 5 V and *V*_D_ of 18 V for 1 msec.

### Electrical Characterization

A B1500A semiconductor parameter analyzer (Keysight) was adopted to measure the electrical characteristics.

### SEM and TEM Analysis

SEM images were captured using a Magellan 400 field emission SEM (FEI Company), and TEM images were captured using HD‐2300A FE‐STEM (Hitachi High‐Tech Corporation).

### Thermal Simulation

The temperature distribution was calculated by COMSOL Multiphysics 5.3a in the heat transfer mode. The thermal diffusion and temperature distribution of the overall structure were calculated by the following heat‐transfer equation:

(2) $$\rho C\frac{\partial T}{\partial t}-\nabla \cdot \;\left(k\nabla T\right)=\;Q$$

Here, ρ is the density, *C* is the heat capacity, *T* is the temperature, *t* is the time, *k* is the thermal conductivity, and *Q* is the heat flux. Each value of the temperature‐dependent parameters (ρ, *C*, *k*) for a specific material (sc‐Si, poly‐Si, SiO_2_) was taken from the COMSOL material library.

For the ELA simulation, the laser energy was converted to the thermal energy for the heat source using the following equation:

(3) $$Q\;\left(x,y\right)=\left(1-R\right)\;I_{0}\alpha_{C}e^{-\alpha_{c}y}$$

where *Q*(*x*,*y*) is the heat flux exponentially decayed with a film depth of *y*, *R* is the reflectance, *I*_0_ is the fluence of the irradiated laser, and *α*_C_ is the absorption coefficient. In a cross‐sectional view of *Q*(*x*,*y*), *x* is the lateral position for scanning and *y* is the depth location for laser attenuation. It should be noted that the thickness of each layer (poly‐Si, ILD, sc‐Si, BOX) was reflected in the simulation.

For the ETA simulation, the measured *I*_punch_ was used as the input variable to the channel in order to obtain the simulated temperature profile. In addition, dimensions of the fabricated 1TFT‐synapse (*T*_Si_, *W*_G_, *L*_G_, gate dielectric thickness, gate poly‐Si thickness) were reflected.

### Hardware‐Based Simulation and Software‐Based Simulation

Hardware‐based simulations, which support circuit simulations for kernel operation, were conducted using LTspice software (Analog Devices). The software‐based simulations, which facilitate image classification of ASL, were conducted with PyTorch, a machine learning library, and Python 3.7 with NVIDIA's GeForce RTX 3090 GPU. The Video S1 (Supporting Information) was made using Matplotlib from the Python visualization library.

## Conflict of Interest

The authors declare no conflict of interest.

## Supporting information

Supporting Information

Supplemental Video 1

## Data Availability

The data that support the findings of this study are available from the corresponding author upon reasonable request.
