# Supplementary material for: 3D Neuromorphic Hardware with Single Thin‐Film Transistor Synapses Over Single Thin‐Body Transistor Neurons by Monolithic Vertical Integration
Source: Adv Sci (Weinh). 2023 Sep 15;10(30):2302380. doi: 10.1002/advs.202302380 (PMC10602577; doi:10.1002/advs.202302380)
Supplement: Supplementary file 1 — Supporting Information [file ADVS-10-2302380-s001.pdf]

ADVANCED SCIENCE Open Access

Supporting Information

for *Adv. Sci.*, DOI 10.1002/advs.202302380

3D Neuromorphic Hardware with Single Thin-Film Transistor Synapses Over Single Thin-Body Transistor Neurons by Monolithic Vertical Integration

Joon-Kyu Han, Jung-Woo Lee, Yeeun Kim, Young Bin Kim, Seong-Yun Yun, Sang-Won Lee, Ji-Man Yu, Keon Jae Lee, Hyun Myung and Yang-Kyu Choi**

Supporting Information

3D neuromorphic hardware with single thin-film transistor synapses over single thin-body transistor neurons by monolithic vertical integration

Joon-Kyu Han†, Jung-Woo Lee†, Yeeun Kim†, Young Bin Kim†, Seong-Yun Yun, Sang-Won Lee, Ji-Man Yu, Keon Jae Lee, Hyun Myung and Yang-Kyu Choi**

J.-K. Han
Department of Materials Science and Engineering and Inter-university Semiconductor Research Center, College of Engineering, Seoul National University, Seoul, 08826, Republic of Korea

J.-W. Lee, Y. Kim, S.-Y. Yun, S.-W. Lee, J.-M. Yu, H. Myung, Y.-K. Choi
School of Electrical Engineering, Korea Advanced Institute of Science and Technology (KAIST), 291 Daehak-ro, Yuseong-gu, Daejeon 34141, Republic of Korea

J.-W. Lee
SK Hynix Inc., Icheon 17336, Republic of Korea

Y. B. Kim, K. J. Lee
Department of Materials Science and Engineering, Korea Advanced Institute of Science and Technology (KAIST), 291 Daehak-ro, Yuseong-gu, Daejeon 34141, Republic of Korea

†These authors equally contributed to this work.
E-mail: ykchoi@ee.kaist.ac.kr and hmyung@kaist.ac.kr

Supplementary Figures

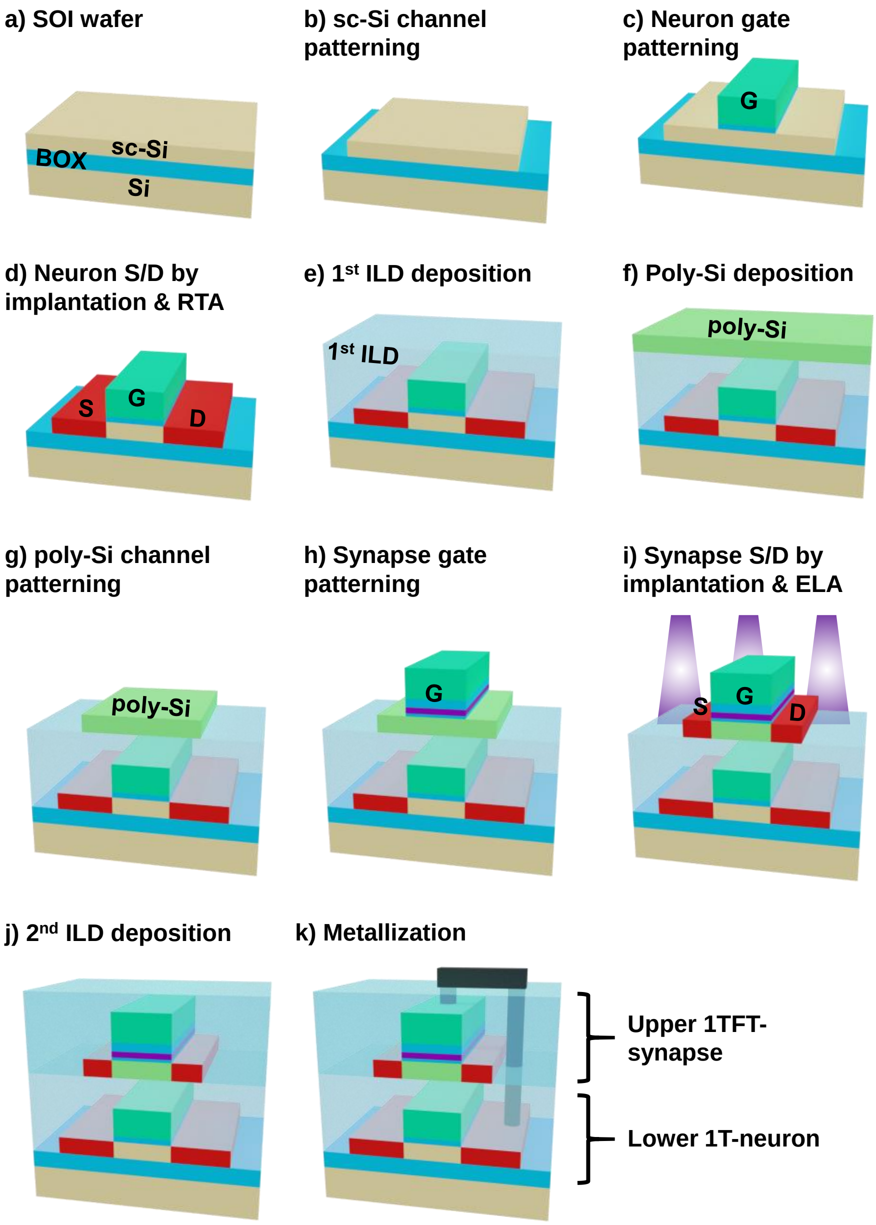

Figure S1. Schematic illustration of the fabrication procedure. a) SOI bare wafer as the starting material. **b**) Neuron sc-Si channel patterning. **c**) Neuron gate patterning for 1T-neuron at the bottom. **d**) Neuron source/drain (S/D) doping by implantation and RTA. **e**) 1st ILD deposition. **f**) Synapse poly-Si deposition. **g**) Synapse poly-Si channel patterning. **h**) Synapse gate patterning for 1TFT-synapse at the top. **i**) Synapse S/D doping by implantation and ELA. **j**) 2nd ILD deposition. **k**) Metallization to interconnect the underlying 1T-neuron and the superjacent 1TFT-synapse. Detailed explanations of the fabrication procedure are provided in Methods of the main manuscript.

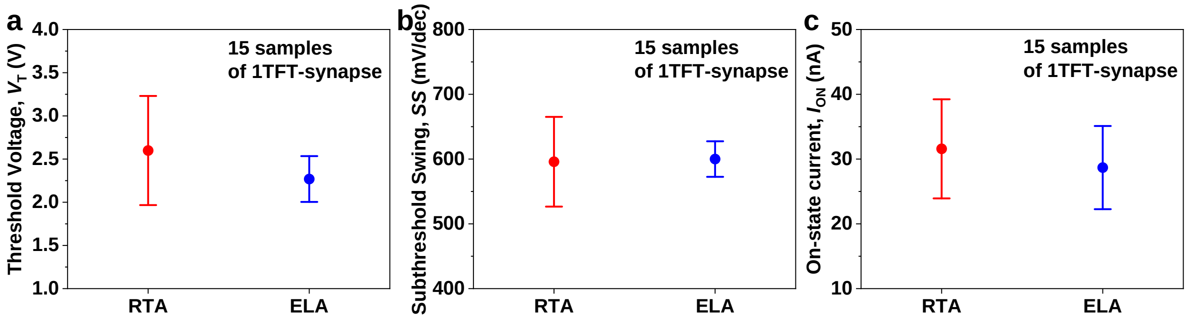

Figure S2. Comparison of the measured electrical properties of the fabricated 1TFT-synapse between the control group (with RTA) and the experimental group (with ELA). **a**) Comparison of the threshold voltage (V_T), **b**) Comparison of the subthreshold swing (*SS*), **c**) Comparison of the on-state current (I_{ON}). The electrical properties were similar in each case.

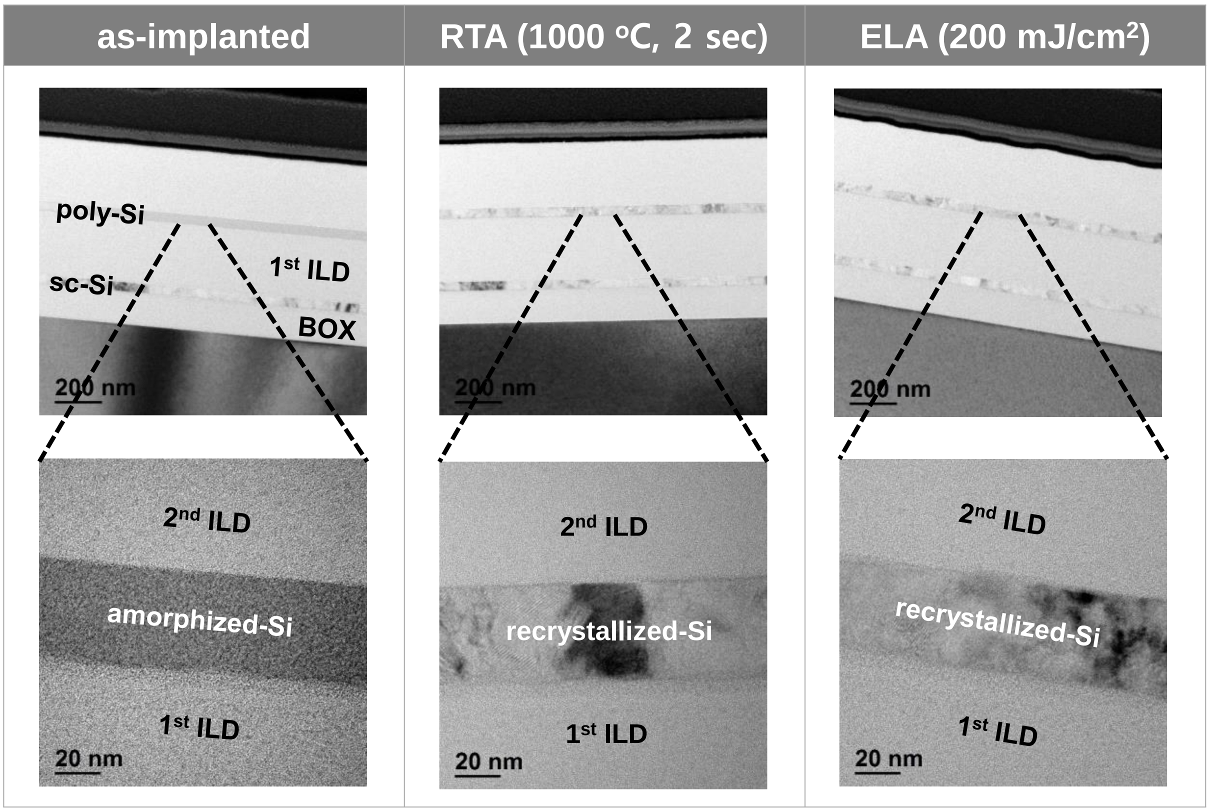

Figure S3. TEM images of poly-Si after S/D implantation, rapid thermal annealing (RTA) and excimer laser annealing (ELA). An amorphous phase was observed after ion implantation for S/D doping. The poly-crystalline phase was confirmed after the RTA and ELA processes.

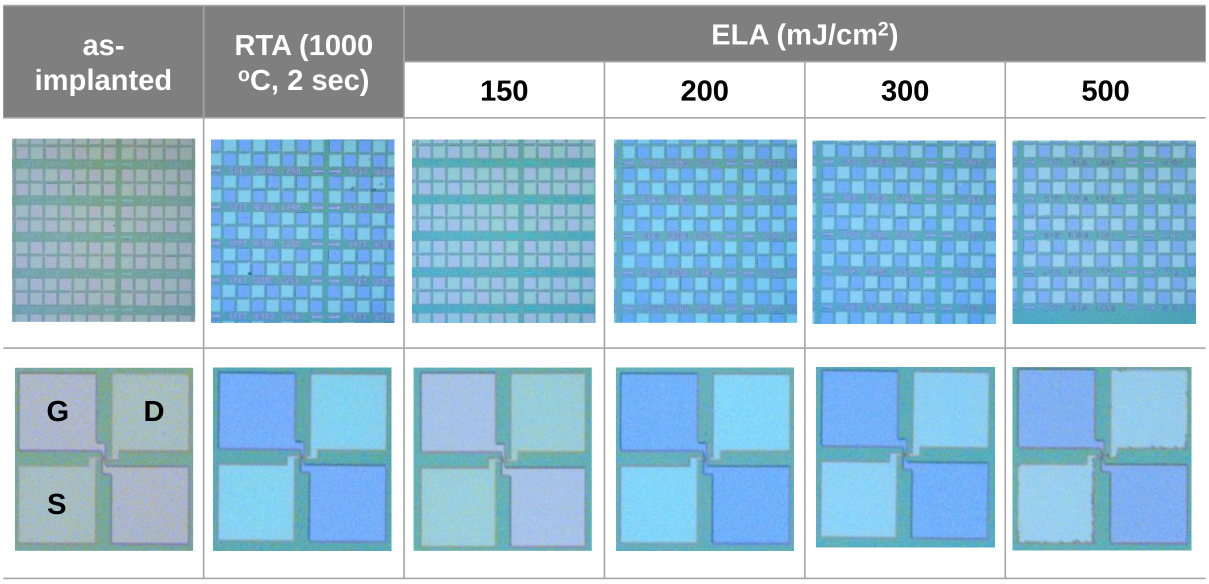

Figure S4. Optical images of the 1TFT-synpase after S/D implantation, rapid thermal annealing (RTA) and excimer laser annealing (ELA). Color change of S/D pads for probing after ELA with laser fluence of 200 mJ/cm^2 for 25 nsec. The colors of the S/D regions appear similar between the control group (with RTA) and the experimental group (with ELA) due to recrystallization. However, laser fluence of 300 mJ/cm^2 for 25 nsec caused the 1TFT-synpase to be destroyed due to the induced high temperature, which exceeded the melting point of the poly-Si.

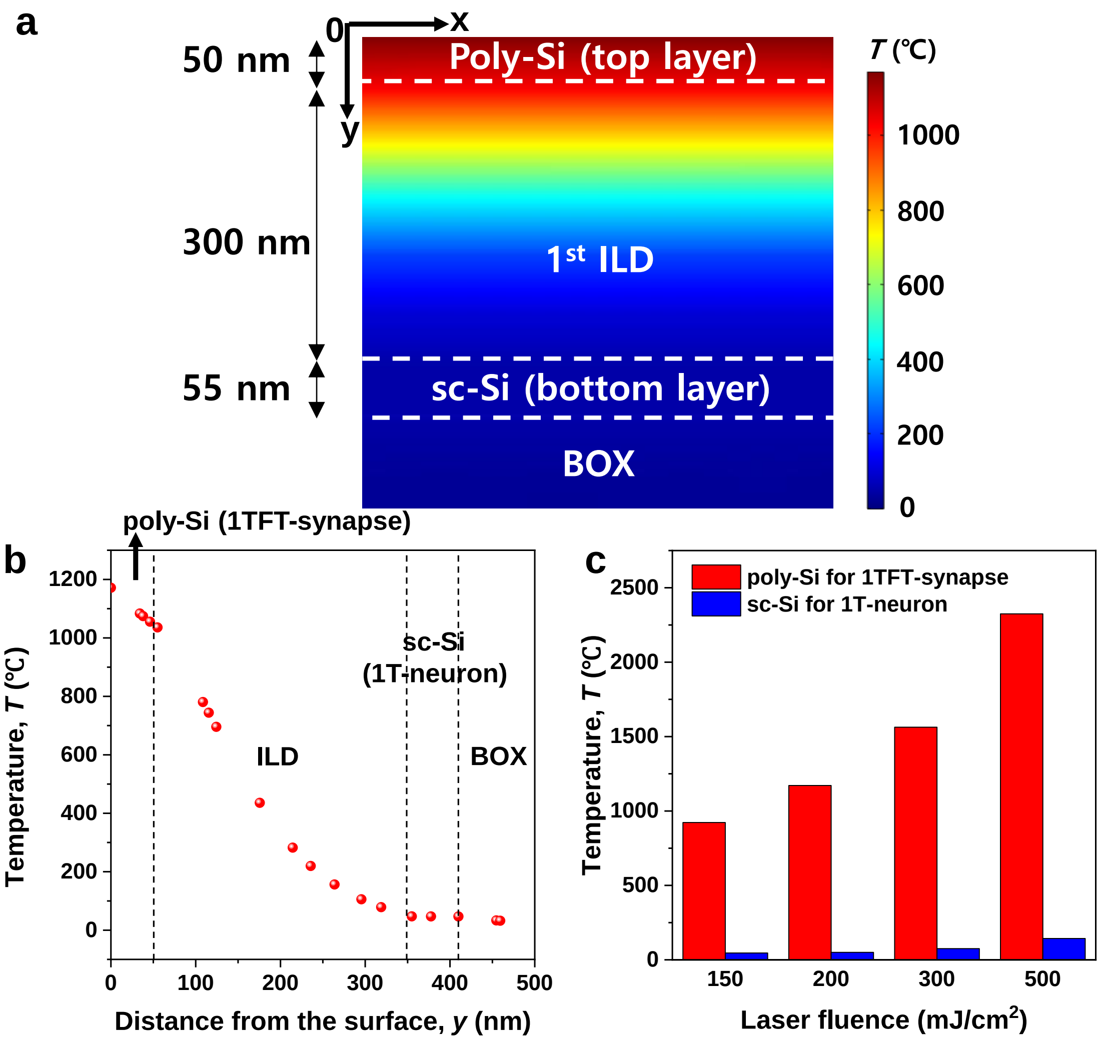

Figure S5. 3D thermal simulation to determine the optimal ELA condition with the aid of COMSOL. a) Cross-sectional 2-D heat distribution profile during ELA. **b**) Extracted temperature along the depth direction (y) from the top surface. **c**) Extracted temperature of the superjacent poly-Si channel and the underlying sc-Si channel for various laser fluences.

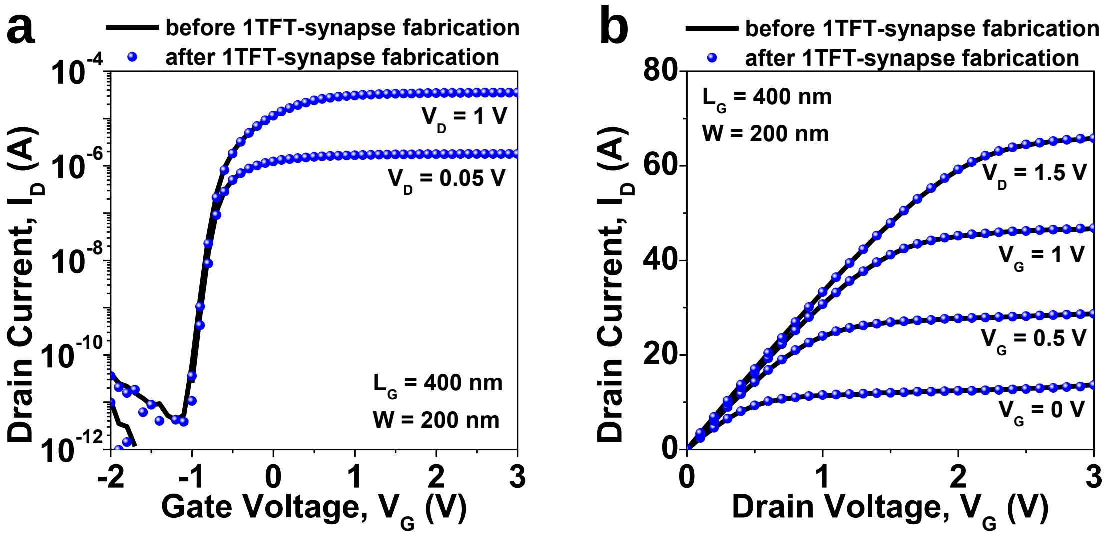

Figure S6. Comparison of the measured electrical characteristics from the fabricated 1T-neuron for two cases: before 1TFT-synpase fabrication at the top and after 1TFT-synpase fabrication. **a**) Transfer characteristic curve (I_D-V_G) and **b**) output characteristic curve (I_D-V_D) of the 1T-neuron. No significant change was observed.

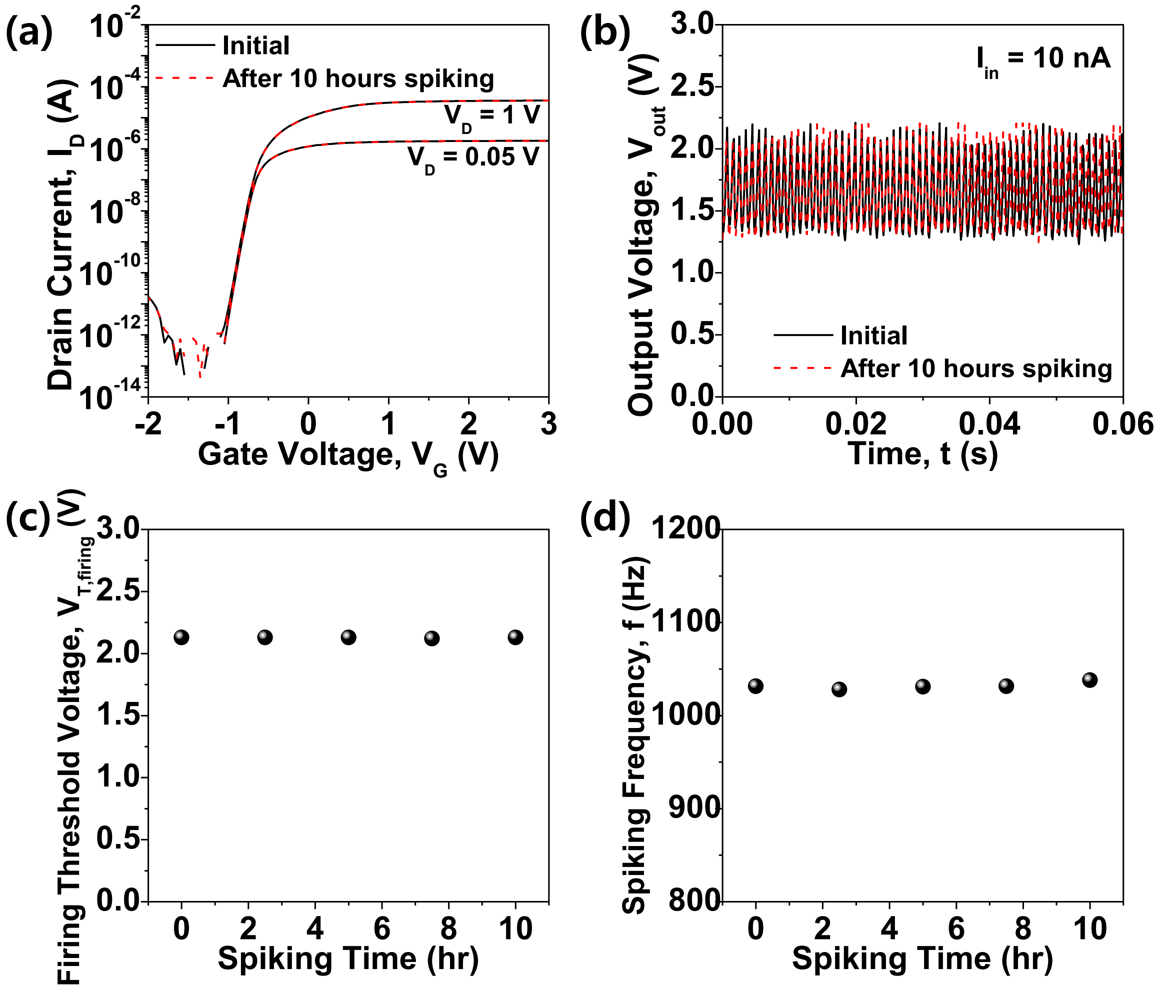

Figure S7. Endurance characteristics of the 1T-neuron. a) Transfer characteristics (I_D-V_G) and **b**) spiking characteristics (V_{out}-t) of the 1T-neuron before and after 10 hours of spiking operations. **c**) Firing threshold voltage ($V_{T,firing}$) and **d**) spiking frequency (f) of the 1T-neuron as a function of spiking time.

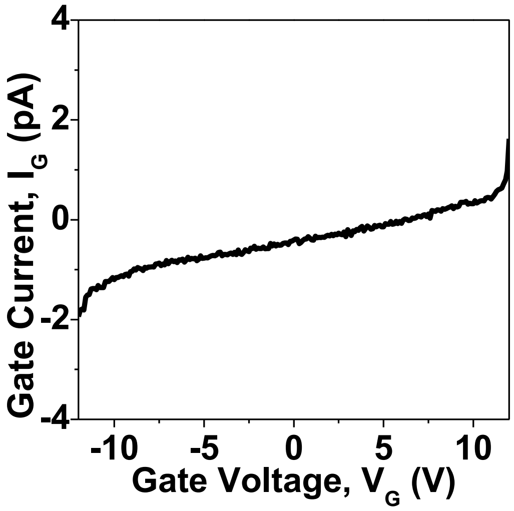

Figure S8. Gate leakage current (I_G) depending on the gate voltage (V_G) of the 1TFT-synapse. A small amount of energy was consumed for the weight update by potentiation and depression due to the relatively low pA of I_G.

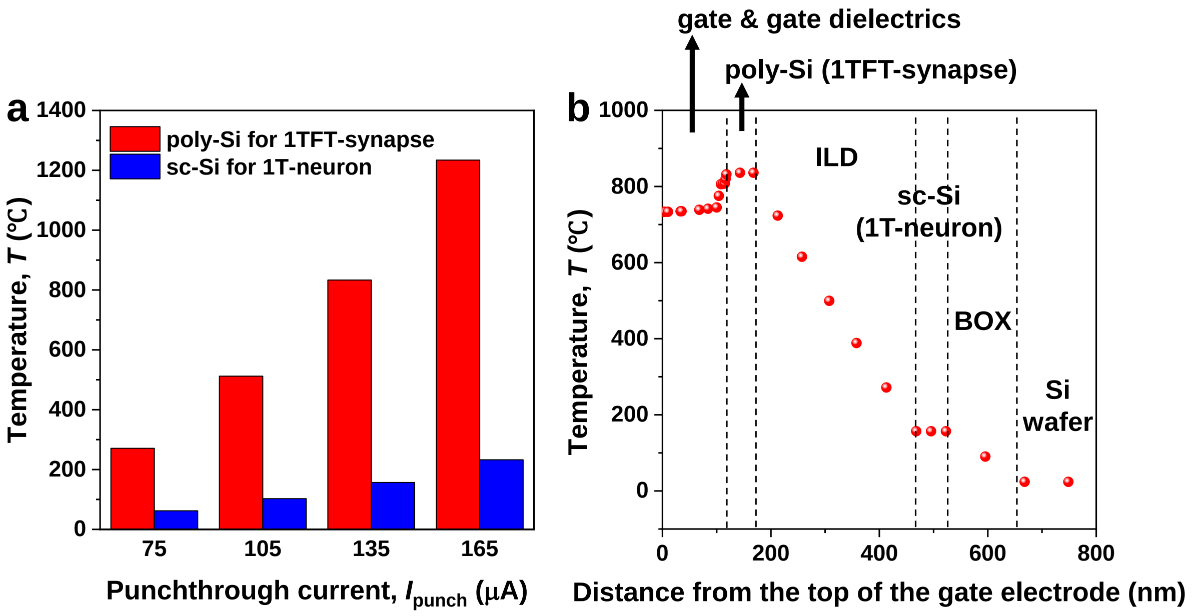

Figure S9. 3D thermal simulation to find the optimal Joule heating condition with the aid of COMSOL. a) Extracted temperature at the poly-Si layer of the 1TFT-synapse and sc-Si of the 1T-neuron for various punchthrough currents (I_{punch}). **b**) Extracted temperature along the depth direction from the top surface of the 1TFT-synapse gate.

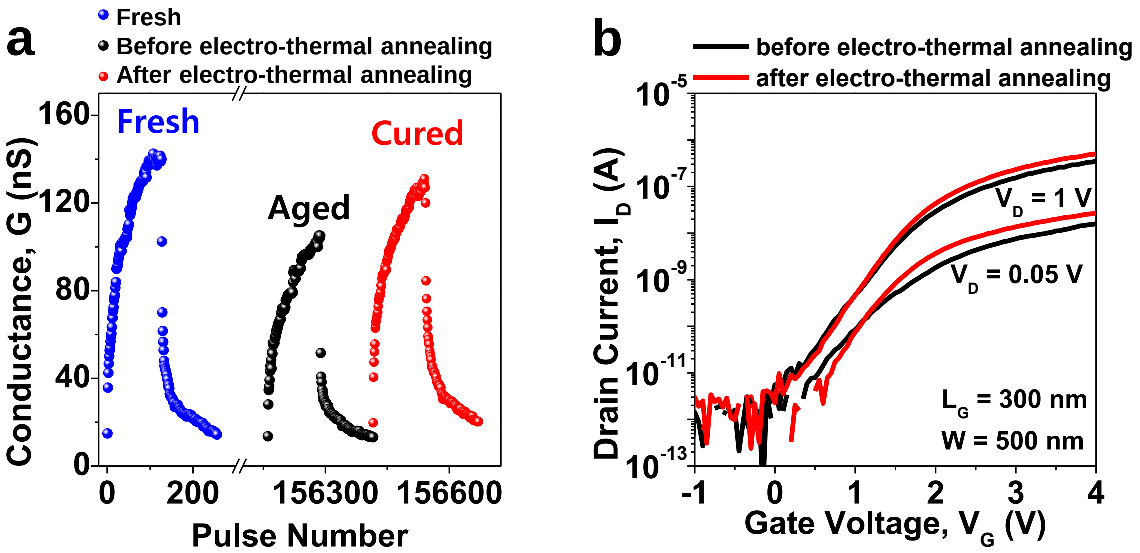

Figure S10. Comparison of the measured electrical characteristics of the 1TFT-synapse before and after Joule heat annealing. a) Potentiation/depression (P/D) curves before and after Joule heat annealing. P/D curves were extracted after 156,160 pulses during the endurance test shown in Figs. 3c and 3d in the main manuscript. Degraded conductance (*G*) by cyclic operation was recovered. **b**) I_D-V_G curve before and after Joule heat annealing. The subthreshold swing (*SS*) and on-state current (I_{ON}) were improved by Joule heat annealing.

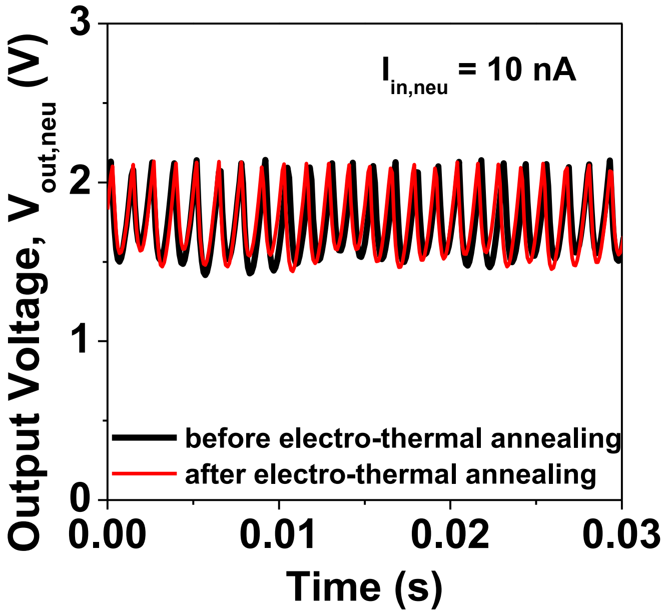

Figure S11. Comparison of the measured spiking characteristics of the underlying 1T-neuron before and after Joule heat annealing applied to the superjacent 1TFT-synapse. The spiking characteristic ($V_{out,neu}$-time) was scarcely changed before and after Joule heat annealing.

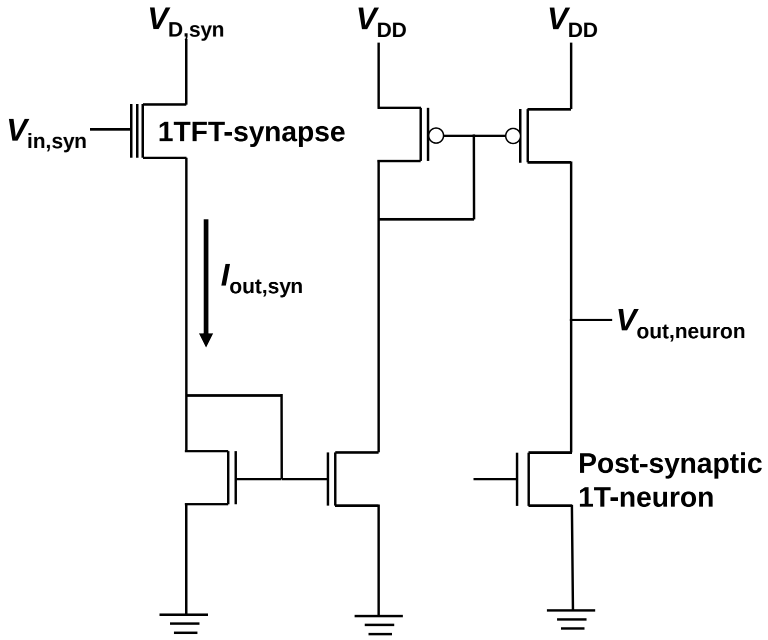

Figure S12. Circuit diagram of the current mirror. A current mirror can be positioned between the 1TFT-synapse and the post-synaptic 1T-neuron as an interface.

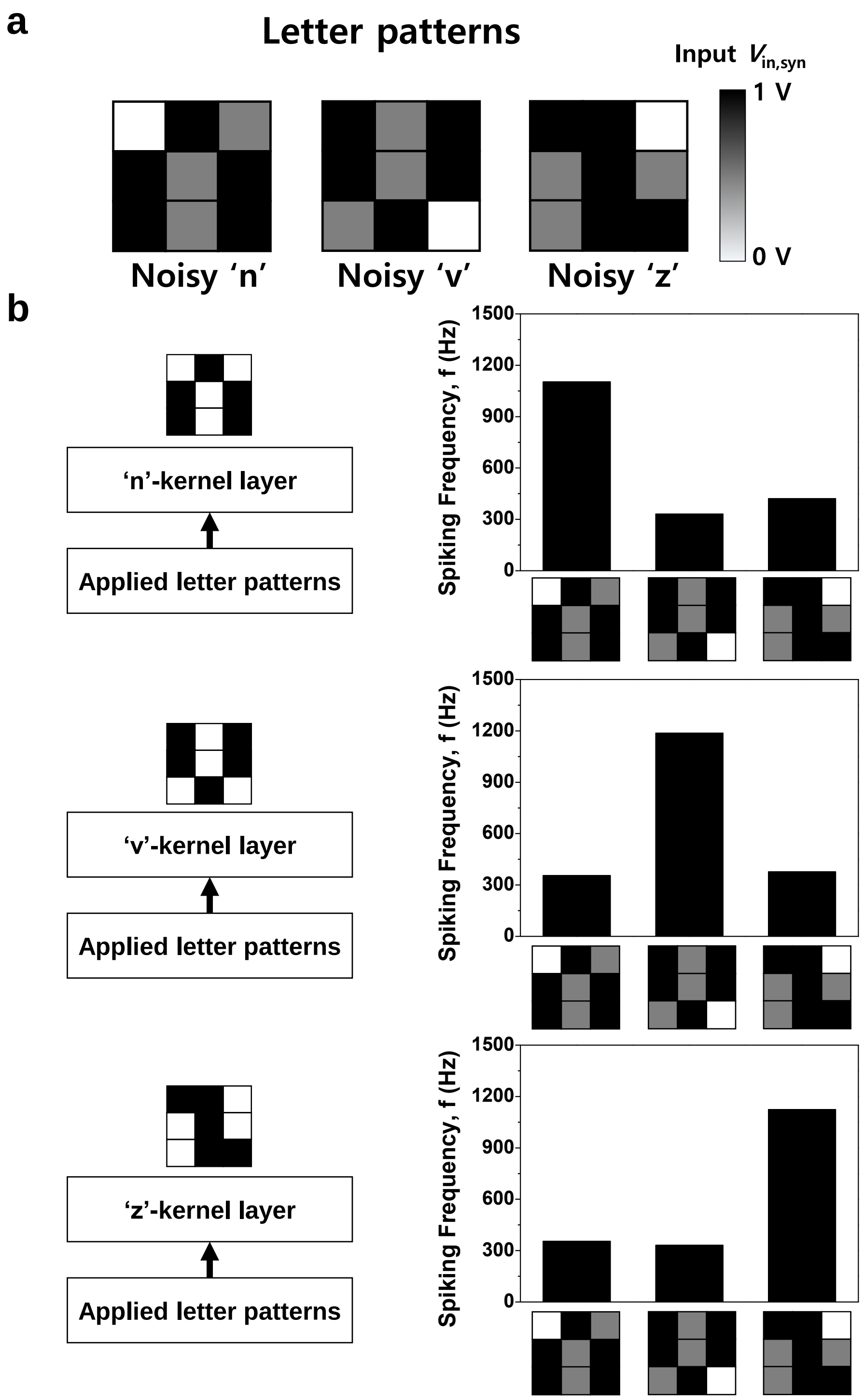

Figure S13. Simulated kernel operation with the LTspice circuit simulator. a) Three letter patterns, 'n', 'v', and 'z', enclosing noisy signals. The pixel intensity corresponds to V_G applied to the 1TFT-synapse ($V_{in,syn}$). A darker shade indicates a higher V_G. **b**) Spiking frequency (*f*) responses to the three image patterns. A highest *f* was found for a matched letter, even in the presence of noise.

Supplementary Tables

1. Device parameters

	Si channel thickness (T_{Si})	Gate width (W_G)	Gate length (L_G)	Gate dielectric thickness	Gate poly-Si thickness
**Top 1TFT-synapse**	50 nm	300 nm	500 nm	3/6/8 nm ($SiO_2/Si_3N_4/SiO_2$)	100 nm
**Bottom 1T-neuron**	55 nm	200 nm	400 nm	5 nm (SiO_2)	100 nm

2. Comparison with previously reported device-level artificial neurons

Ref.	Configuration	Operating voltage	Energy per Spike	CMOS compatibility
S1	Biristor	2.5 V	6 pJ/spike	Full
S2	Planar MOSFET	> 2.5 V	30 pJ/spike	Full
S3	Double-gate MOSFET	6.5 V	950 pJ/spike	Full
S4	Vertical MOSFET	5.5 V	1.4 nJ/spike	Full
S5	NbO_x memristor	~1.1 V	~50 pJ/spike	Partial
	B-Te memristor	~0.7 V	~30 pJ/spike	Partial
	HfO_2 memristor	~0.3 V	~20 pJ/spike	Full
S6	Phase-change memory	> 1 V	30 pJ/spike	Partial
**This work**	**Planar MOSFET**	**2 V**	**19.3 pJ/spike**	**Full**

3. Comparison with previously reported stacked synaptic devices by 3D integration

Ref.	Device structure	# of states	Pulse scheme	g_{max}/g_{min}	Nonlinearity ($\alpha_{pot}/\alpha_{dep}$)	Retention	Endurance	Stacking demonstration	Application
S7	InGaAs FET	128	Varying	6,300	1.22/-1.75 (varying pulse)	> 60,000 sec	> 64,000 pulses (128 states)	-	Deep neural network (DNN)
S8	WO_x memristor	100	Identical	2	Poor	-	> 10^7 cycles (2 states)	Stacked over CMOS circuits	Vector-matrix multiplication (VMM)
S9	$HfAlO_x$ memristor	300	Identical	~30	-	> 10,000 sec (@ 125 °C)	-	Stacked over CMOS circuits	Computing-in-memory (CIM)
**This work**	**Poly-Si TFT**	**128**	**Identical**	**9.55**	**3.70/-2.13 (identical pulse)**	**> 100,000 sec**	**> 205,000 pulses (128 states)**	**Stacked over neuron device**	**Spiking neural network (SNN)**

4. Average and standard deviation of device parameters extracted from different 1T-neurons and 1TFT-synapses.

	Parameter	Average	Standard deviation
1T-neuron (10 devices)	V_T (V)	-0.858	0.0292
	SS (mV/dec)	75.9	6.41
	I_{ON} (μA)	1.51	0.166
	$V_{T,firing}$ (V)	1.94	0.0629
1TFT-synapse (10 devices)	V_T (V)	2.17	0.211
	SS (mV/dec)	598	81.5
	I_{ON}(nA)	21.1	3.99
	g_{max}/g_{min}	6.89	0.349

5. Network configuration used for ASL classification with a simulation

Layer type	Kernel	Output
Input		2@180×240
Average-pooling	4×4	2@45×60
Convolutional	16@C5 (padding = 2)	16@45×60
Average-pooling	2×2	16@23×30
Convolutional	32@C3 (padding = 1)	32@23×30
Average-pooling	2×2	32@12× 15
Fully-connected		512
Output		24

6. Extracted synaptic parameters of P/D characteristics for ASL classification

	g_{max}	g_{min}	α_{pot}	α_{dep}
**Fresh**	131	19.7	4.39	-2.55
**After 204,800 update pulses without ETA**	64.5	9.68	2.08	-1.80
**After 204,800 update pulses with ETA**	126	18.8	4.74	-2.77

Supplementary Note 1

1. Details of ASL classification

(1) Data processing**:** The ASL dataset was collected using a dynamic vision sensor (DVS).[S10] It contains information about the position (x,y) where the event occurred and the time it occurred. It also includes the polarity of the event (ON or OFF). Because each sample had a different length, each was cropped to an identical length of 100 msec. For the training dataset, 84,000 samples were randomly selected from the full dataset, and the remaining 16,800 samples were used for testing. All of the images had an original full size of 180×240 pixels.

(2) Network structure: The spiking-CNN was composed of two spiking convolutional layers, three average pooling layers, and two fully connected layers. Because the ASL dataset had 24 classes, the output layer had 24 output spiking neurons. The output spiking neuron generating the largest number of spikes was considered as the inference result; *i.e.*, the rule of winner-take-all was used.

(3) Training method: Because the spiking-CNN utilizes a non-differentiable spike function, the traditional backpropagation that requires the differentiation of the spike function cannot be applied. Thus, backpropagation method using spike layer error reassignment in time (SLAYER) was used as an alternative.[S11] When using this method, the derivative of the spike function is approximated by the probability distribution function (PDF) for a change of the spike state, as follows:

$\rho(t) = \exp(-\beta \cdot |u(t)-V_T|)/\alpha$ (S1),

where α and β are the parameters to determine the shape of the PDF, $u(t)$ is the membrane potential at time t, and V_T is the firing threshold voltage. Note that the spike state of the neuron changes between the two states, from *not-fire* to *fire* or from *fire* to *not-fire* at t. The $\rho(t)$ decays exponentially according to $|u(t)-V_T|$ at t. For the loss function, the difference between the number of output spikes generated from the input and the desired number of spikes was used[S11].

(4) Supporting video: Supporting video was created to show the successful classification of the ASL dataset. On the left side, the input spikes of each coordinate were visualized, where ON event was colored green and OFF event was colored blue. On the right side, the number of output spikes generated from each output neuron over time was visualized using a bar graph. The bar with the most spikes was colored differently to check the classification result. As a result, the output neuron that generates the largest number of spikes matched well with the input image. For visualization, Matplotlib, a python visualization library, was used.

References

[S1] J.-W. Han, M. Meyyappan, *IEEE Electron Device Lett.* **2018**, *39*, 1457.

[S2] J.-K. Han, M. Seo, W.-K. Kim, M.-S. Kim, S.-Y. Kim, M.-S. Kim, G.-J. Yun, G.-B. Lee, J.-M. Yu, Y.-K. Choi, *IEEE Electron Device Lett.* **2020**, *41*, 208.

[S3] J.-K. Han, M. Seo, J.-M. Yu, Y.-J. Suh, Y.-K. Choi, *IEEE Electron Device Lett.* **2020**, *41*, 1157.

[S4] J. Han, J. Oh, J. Yu, S. Choi, Y. Choi, *Small* **2021**, *17*, 2103775.

[S5] D. Lee, M. Kwak, K. Moon, W. Choi, J. Park, J. Yoo, J. Song, S. Lim, C. Sung, W. Banerjee, H. Hwang, *Adv. Electron. Mater.* **2019**, *5*, 1800866.

[S6] T. Tuma, A. Pantazi, M. Le Gallo, A. Sebastian, E. Eleftheriou, *Nat. Nanotechnol.* **2016**, *11*, 693.

[S7] S. K. Kim, Y. Jeong, P. Bidenko, H.-R. Lim, Y.-R. Jeon, H. Kim, Y. J. Lee, D.-M. Geum, J. Han, C. Choi, H. Kim, S. Kim, *ACS Appl. Mater. Interfaces* **2020**, *12*, 7372.

[S8] F. Cai, J. M. Correll, S. H. Lee, Y. Lim, V. Bothra, Z. Zhang, M. P. Flynn, W. D. Lu, *Nature Electron.* **2019**, *2*, 290.

[S9] Y. Li, J. Tang, B. Gao, J. Yao, Y. Xi, Y. Li, T. Li, Y. Zhou, Z. Liu, Q. Zhang, S. Qiu, Q. Li, H. Qian, H. Wu, *2021 IEEE International Electron Devices Meeting (IEDM)* **2021**, 21.

[S10] Y. Bi, A. Chadha, A. Abbas, E. Bourtsoulatze, Y. Andreopoulos, *Proc. IEEE Int. Conf. Comput. Vis.* **2019**, 491.

[S11] S. B. Shrestha, G. Orchard, *Adv. Neural Inf. Process. Syst.***2018**, 31.
